# Impact of COVID-19 Prevention and Control on the Influenza Epidemic in China: A Time Series Study

**DOI:** 10.34133/2022/9830159

**Published:** 2022-11-01

**Authors:** Zirui Guo, Li Zhang, Jue Liu, Min Liu

**Affiliations:** ^1^Department of Epidemiology and Biostatistics, School of Public Health, Peking University, Beijing, China; ^2^Beijing Center for Disease Prevention and Control, Beijing, China

## Abstract

*Background*. COVID-19 prevention and control measures might affect influenza epidemic in China since the nonpharmaceutical interventions (NPIs) and behavioral changes contain transmission of both SARS-CoV-2 and influenza virus. We aimed to explore the impact of COVID-19 prevention and control measures on influenza using data from the National Influenza Surveillance Network.*Methods*. The percentage of influenza-like illness (ILI%) in southern and northern China from 2010 to 2022 was collected from the National Influenza Surveillance Network. Weekly ILI% observed value from 2010 to 2019 was used to calculate estimated annual percentage change (EAPC) of ILI% with 95% confidence intervals (CIs). Time series analysis was applied to estimate weekly ILI% predicted values in 2020/2021 and 2021/2022 season. Impact index was used to explore the impact of COVID-19 prevention and control on influenza during nonpharmaceutical intervention and vaccination stages.*Results*. China influenza activity was affected by the COVID-19 pandemic and different prevention and control measures during 2020-2022. In 2020/2021 season, weekly ILI% observed value in both southern and northern China was at a low epidemic level, and there was no obvious epidemic peak in winter and spring. In 2021/2022 season, weekly ILI% observed value in southern and northern China showed a small peak in summer and epidemic peak in winter and spring. The weekly ILI% observed value was generally lower than the predicted value in southern and northern China during 2020-2022. The median of impact index of weekly ILI% was 15.11% in north and 22.37% in south in 2020/2021 season and decreased significantly to 2.20% in north and 3.89% in south in 2021/2022 season.*Conclusion*. In summary, there was a significant decrease in reported ILI in China during the 2020-2022 COVID-19 pandemic, particularly in winter and spring. Reduction of influenza virus infection might relate to everyday Chinese public health COVID-19 interventions. The confirmation of this relationship depends on future studies.

## 1. Introduction

Influenza is an acute respiratory infectious disease caused by influenza virus that can cause a high burden and severe seasonal epidemics as well as pandemics. Influenza virus is highly contagious, and symptoms can run from mild to severe, leading to increased morbidity and mortality in high-risk populations, and was one of a major cause of the healthcare service use globally [1, 2]. Influenza is a public health threat and one of the most thoroughly monitored diseases in the world. In most cases, vaccination provides safe and effective protection against seasonal influenza [1].

In December 2019, an outbreak of Coronavirus Disease 2019 (COVID-19) occurred in Wuhan, Hubei Province, China [3]. It was declared a pandemic by the World Health Organization (WHO) on 11th March 2020, and there were more than 562 million confirmed cases of COVID-19 worldwide, including 63 million deaths as of 20th July 2022 [4]. During the fight against COVID-19, nonpharmaceutical interventions (NPIs) and vaccination were used to prevent and control the community transmission of COVID-19, and the local policies varied depending on the severity of the COVID-19 outbreak.

Influenza-like illness (ILI) usually presents as fever, dry cough, and fatigue. Since the majority of COVID-19 patients exhibit symptoms, it is difficult to distinguish COVID-19 patients from influenza based on symptoms alone [5]. The transmission mechanism and seasonal coincidence of severe acute respiratory syndrome coronavirus 2 (SARS-CoV-2) are similar to those of influenza viruses [6]. Moreover, cases of coinfection with influenza and SARS-CoV-2 have been reported in some countries [6– 8]. The cotransmission of SARS-CoV-2 and influenza may increase the burden of respiratory disease [9].

Respiratory diseases occur frequently in winter and spring. The risk of COVID-19, influenza, and other respiratory infections’ cocirculation exists [5], making prevention work more complicated and difficult. Public health measures have been deemed effective in reducing the burden of influenza outbreaks [10]. Preliminary studies have reported that COVID-19 NPIs and behavioral changes could have affected transmission dynamics of influenza and other respiratory diseases [10, 11], but the evidence only supported the impact in early stage of COVID-19 pandemic. However, no study has systematically explored the long-term relationship between Chinese prevention and control measures and influenza during the COVID-19 pandemic, especially for the impact under different prevention and control levels till now.

In our study, we used data from the National Influenza Surveillance Network of China to explore the impact of the COVID-19 pandemic on reported influenza cases in China and the impact of COVID-19 prevention and control measures on influenza prevention.

## 2. Methods

### 2.1. Data Source and Definition of Influenza

The data of influenza or ILI outbreaks from the 14th week of 2010 to the 13th week of 2022 were collected from the National Influenza Surveillance Network of Chinese National Influenza Center. The National Influenza Surveillance Network of the Chinese National Influenza Center received influenza data reported by the Chinese National Influenza Surveillance Information System (CNISIS) and network laboratories in 31 provinces of mainland China. Currently, 554 sentinel hospitals and 410 laboratories are included in the Chinese National Influenza Surveillance Network. The standardized surveillance protocols and operating procedures used by the Chinese National Influenza Center have been described elsewhere [12]. 

According to the *Technical Guidelines for National Influenza Surveillance* (2017 edition) [12], ILI was defined as fever (temperature≥38°C) symptom with cough or sore throat. The onset of fever should be within this acute fever course, and body temperature determination was detected by patients themselves or medical institutions. Percentage of visits for $\text{influenza}-\text{like}\;\text{illness}\;\left(\text{ILI}\%\right)=\text{Number}\;\text{of}\;\text{ILI}/\left(\text{total}\;\text{visit}\;\text{number}\;\text{of}\;\text{outpatient}\;\text{or}\;\text{emergency}\;\text{department}\right)\times 100\%$. In China, weekly virological and ILI data, in influenza sentinel surveillance, are systematically collected and used as a proxy of influenza activity [11]. 

The 14th week of each year is the beginning week of the surveillance year, and the 13th week of the following year is the end of the monitoring surveillance year [12]. The surveillance year was classified into two periods based on the disease characteristics. Influenza nonepidemic season was defined as the period from the 14th to 39th week, which from early April to late September. Influenza epidemic season was defined as period from the 40th week to the 13th week of the following year, which from early October to late March [13]. 

### 2.2. Definition of Stages of COVID-19 Prevention and Control in China

We divided the period 2020-2022 into seven stages based on COVID-19 prevention and control events during the pandemic (Table 1). The stage division was mainly based on *Fighting Covid-19 China in Action* [14] published on June 7th, 2020 by information office of the state council and related studies [15]. Sever stages are shown in Table 1. Stage VI was started by January 9th 2021 when China announced the launch of COVID-19 vaccination for all free of charge [16]. From August 2021, China's epidemic prevention and control entered the “dynamic COVID-zero” phase of whole-chain precise prevention and control [15], which was stage VII. 

**Table 1:** The stage division of COVID-19 prevention and control in China.

Stage	Date	Stage division
Stage I	December 27th, 2019-January 19th, 2020	Swift response to the public health emergency
Stage II	January 20th-February 20th, 2020	Initial progress in containing the virus
Stage III	February 21th-march 17th, 2020	Newly confirmed domestic cases on the Chinese mainland drop to single digits
Stage IV	March 18th-April 28th, 2020	Wuhan and Hubei—an initial victory in a critical Battle
Stage V	Since April 29th, 2020	Ongoing prevention and control
Stage VI	January 9th 2021	China announced the launch of COVID-19 vaccination for all free of charge
Stage VII	From August 2021	“Dynamic COVID-zero” phase of whole-chain precise prevention and control

### 2.3. Statistical Analysis

#### 2.3.1. Calculation of Estimated Annual Percentage Change

This study focused on the impact of COVID-19 prevention and control measures on the influenza. We calculated the mean weekly ILI% observed value from 2010 to 2019 in southern and northern China in order to compare with the observed value from 2020 to 2022. We used weekly ILI% observed value from 2010 to 2019 in southern and northern China to calculate the weekly estimated annual percentage change (EAPC) of ILI% with 95% confidence intervals (CIs) for each calendar week to quantify the ILI% trends, with the 10 observed values from 2010 to 2019. Durbin Watson (DW) test was done to examine the autocorrelation, and the DW statistic was shown in the supplementary table. No autocorrelation was identified. EAPC is a summary and widely used measure of the infectious disease rates over a specified time interval. A regression line was fitted to the natural logarithm of the ILI%, that is,y=α+βx+ε, where $y=\ln\;\left(\text{ILI}\%\right)$ and x is the calendar year. EAPC was calculated as $100\times \left(e^{\beta }-1\right)$, and its 95% CIs were also calculated to measure the temporal trend of ILI% [17]. The ILI% was deemed to be in an increasing trend if the EAPC estimation, and the lower boundary of its 95% CI were both >0. In contrast, the ILI% showed a decreasing trend if the EAPC estimation and the upper boundary of its 95% CI were both <0 [18].

#### 2.3.2. Time Series Analysis of Weekly ILI%

Time series analysis was applied to estimate weekly ILI% predicted value in 2020/2021 and 2021/2022 season, to compare the predicted value and observed value of weekly ILI%. Weekly ILI% predicted value in $2020/2021\;\text{season}=\text{ILI}{\%}_{2019\;\text{observed}\;\text{value}}\times \left(1+\text{EAPC}\right)$. Weekly ILI% predicted value in $2021/2022\;\text{season}=\text{ILI}{\%}_{2019\;\text{observed}\;\text{value}}\times {\left(1+\text{EAPC}\right)}^{2}$. The median and interquartile range (IQR) (75% quantile -25% quantile) of the predicted value and observed value were calculated to quantify their discrete degree. T test was used to compare the means of the observed value and predicted value.

#### 2.3.3. Calculation of Impact Index

We developed impact index to explore the impact of COVID-19 prevention and control on influenza during the nonpharmaceutical intervention and vaccination stages. Impact index of weekly $\text{ILI}\%=\left(\left(\text{ILI}{\%}_{\text{predicted}\;\text{value}}-\text{ILI}{\%}_{\text{observed}\;\text{value}}\right)/\text{ILI}{\%}_{\text{predicted}\;\text{value}}\right)\times 100\%$. The impact index >0 suggested a positive prevention influence on influenza infection may exist in that week. The impact index <0 suggested a negative prevention influence on influenza infection may exist in that week. T test was used to compare the impact between the epidemic and nonepidemic seasons, as well as the same period between 2020/2021 and 2021/2022 seasons.

STATA 16.0 was used for statistical analysis. Statistical significance was set at a level of p<0.05.

## 3. Results

### 3.1. Epidemic Trends and Characteristics of Influenza in Southern and Northern China

**Figure 1:**
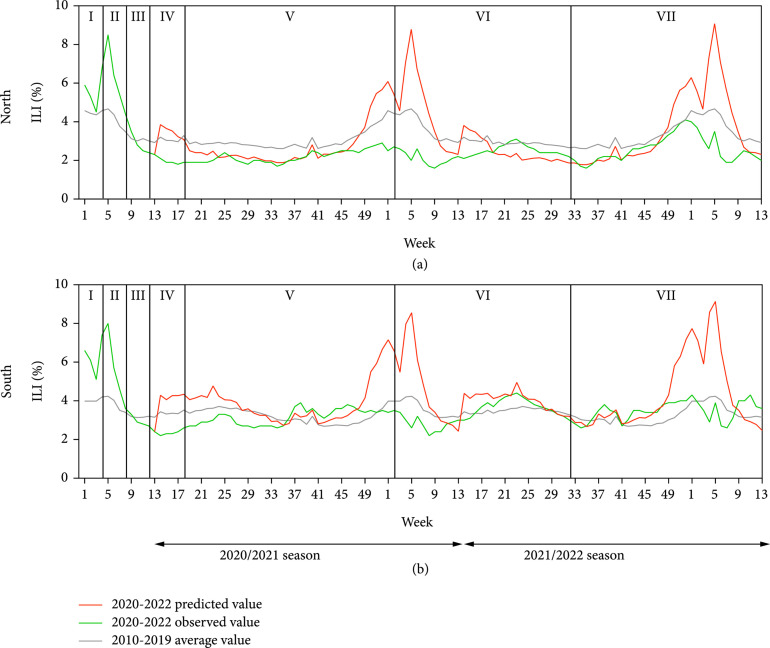
The curves of observed and predicted value of percentage of influenza-like illness (ILI%) in southern and northern China from 2020 to 2022. (a) The curves of the observed and predicted values of ILI% in northern China. (b) The curves of observed and predicted values of ILI% in southern China. Note: stage I: swift response to the public health emergency. Stage II: initial progress in containing the virus. Stage III: newly confirmed domestic cases on the Chinese mainland drop to single digits. Stage IV: Wuhan and Hubei—an initial victory in a critical battle. Stage V: ongoing prevention and control. Stage VI: free COVID-19 vaccination for all of charge. Stage VII: “dynamic COVID-zero” phase of whole-chain precise prevention and control.

Based on data from the National Influenza Surveillance Network, the mean weekly ILI% observed value in southern and northern China from 2010 to 2019 was shown in Figure 1. The weekly ILI% observed value presented obvious epidemic characteristics in winter and spring from the 40th week to the 13th week of the next year, while it also presented a small peak in summer in southern China from the 14th week to the 30th week. EAPC of weekly ILI% observed value in southern and northern China from 2010 to 2019 was shown in Table S1. 

In different stages of COVID-19 prevention and control in China, weekly ILI% observed value curves of southern and northern China in 2020/2021 season were flat (Figure 1). The range of weekly ILI% observed value was 1.6-2.9% with a median of 2.0% in northern China and 2.2-3.9% with a median of 3.0% in southern China. During stage I and stage II of COVID-19 prevention and control in China (1-8th week), weekly ILI% observed value in southern and northern China was at high level. In stage III (8-12th week) and stage IV (12-18th week), weekly ILI% observed value in both southern and northern China was at low epidemic level and showed a downward trend. After entering the stage V (18th week), weekly ILI% observed value curves showed an upward trend in southern and northern China, but no obvious epidemic peak was observed in winter and spring. In 2021/2022 season, weekly ILI% observed value showed a small peak in summer and an epidemic peak in winter and spring in southern and northern China. By the 9th week of 2022, weekly ILI% observed value in both southern and northern China showed double peaks in 2021/2022 season and an upward trend in the 8th week. 

### 3.2. Comparison of Weekly ILI% Observed Value and Predicted Value

**Figure 2:**
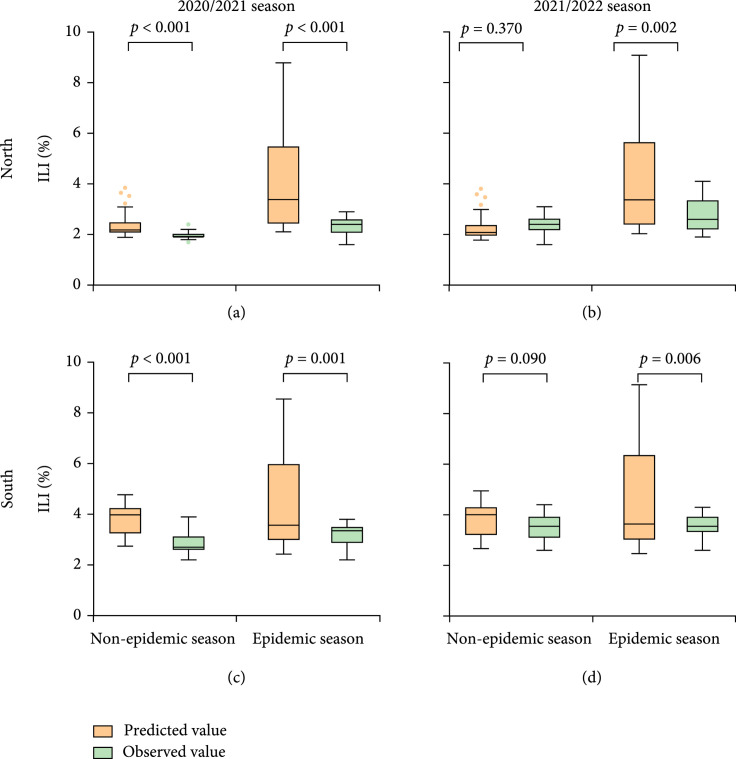
Comparison of weekly ILI% observed value and predicted value from 2020 to 2022. (a) Comparison of weekly ILI% observed value and predicted value between epidemic and nonepidemic seasons from 2020 to 2022 in northern China. (b) Comparison of weekly ILI% observed value and predicted value between epidemic and nonepidemic seasons from 2020 to 2022 in southern China. Note: seasonal influenza nonepidemic season: the 14-39th week. Seasonal influenza epidemic season: the 40-13th week. Box diagram: the middle line is the median. The upper and lower edges of the box are 75% and 25% quantiles; $\text{interquartile}\;\text{range}\;\left(\text{IQR}\right)=75\%\text{quantile}\;\left(Q3\right)-25\%\text{quantile}\;\left(Q1\right)$, $\text{minimum}\;\text{observed}\;\text{value}\;\left(\text{lower}\;\text{edge}\right)=Q1-1.5\;\text{IQR}$, and $\text{maximum}\;\text{observed}\;\text{value}\;\left(\text{upper}\;\text{edge}\right)=Q3+1.5\;\text{IQR}$. Data outliers are presented as dots.

The weekly ILI% predicted value of southern and northern China in 2020/2021 and 2021/2022 season was shown in Table S2 and Table S3, and its curves of weekly ILI% predicted value were shown in Figure 1. A comparison of the weekly ILI% observed and predicted values from 2020 to 2022 was shown in Figure 2. 

In 2020/2021 season, the weekly ILI% observed value and predicted value were different (Figures 2(a) and 2(c)). The mean weekly ILI% observed value was lower than the predicted value in northern nonepidemic season (t=3.927, p<0.001), northern epidemic season (t=4.627, p<0.001), southern nonepidemic season (t=6.375, p<0.001), and southern epidemic season (t=3.476, p=0.001) with a low discrete degree of observed value being small, presenting a low epidemic level in 2020/2021 season. 

In 2021/2022 season, there were also differences between weekly ILI% observed and predicted values (Figures 2(b) and 2(d)). The mean observed value was lower than the predicted value in northern epidemic season (t=3.219, p=0.002) and southern epidemic season (t=2.713, p=0.006), with a low discrete degree of the observed value compared to the predicted value, while the observed value was higher than the predicted value in northern nonepidemic season. Comparing observed value between 2020/2021 and 2021/2022 seasons, a higher discrete degree was observed in 2021/2022 season than the same period of the previous year. 

### 3.3. Comparison of Impact Index of Weekly ILI%

**Figure 3:**
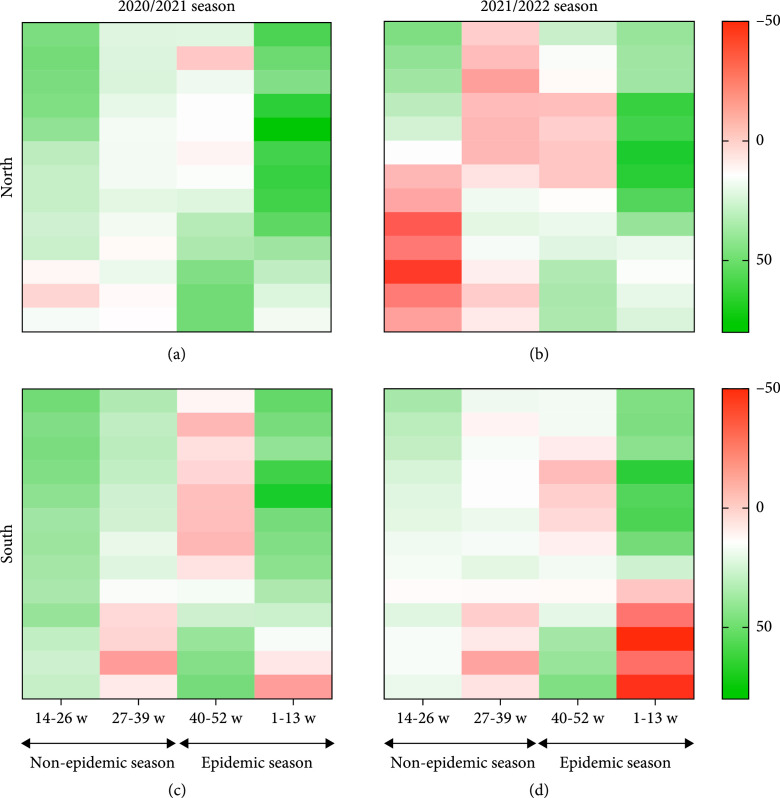
Impact index of epidemic and nonepidemic seasons in northern and southern China from 2020 to 2022. (a) Comparison of impact index of epidemic and nonepidemic seasons in northern China 2020/2021 season. (b) Comparison of impact index of epidemic and nonepidemic seasons in northern China 2021/2022 season. (c) Comparison of impact index of epidemic and nonepidemic seasons in southern China 2020/2021 season. (d) Comparison of impact index of epidemic and nonepidemic seasons in southern China 2021/2022 season. Green means high impact index, and red means low impact index.

In 2020/2021 season, the impact index ranged from -13.48% to 77.24% with a median of 15.11% in northern China (Figure 3(a)), and it ranged from 23.49% to 69.59% with a median of 22.37% in southern China (Figure 3(c)). The impact index between epidemic and nonepidemic seasons was different (t=2.716, p=0.005) in northern China. During 2020/2021 nonepidemic season, impact index showed a downward trend in both southern and northern China. After entering the influenza epidemic season, the impact index showed an upward trend at the beginning and peaked in midseason and then declined during the middle and late seasons. During NPI implementation stage, influenza infections were reduced by 77.24% in northern China compared with predicted value at the 5th week of 2021. A similar pattern was observed in southern China with a 69.59% reduction in influenza infection compared to predicted value. 

In 2021/2022 season, the impact index ranged from -43.78 to 68.97% with a median of 2.20% in northern China (Figure 3(b)), and it ranged from -47.50 to 66.23% with a median of 3.89% in southern China (Figure 3(d)). The impact index between the epidemic and nonepidemic seasons was different (t=4.020, p<0.001) in northern China. A similar pattern of the impact index trend during the nonepidemic and epidemic seasons was observed in northern and southern China. The impact index was lower than that in the same period of the previous year and was less than 0 in some weeks in northern China. The median of impact index decreased significantly compared with the same period in 2020/2021 season in southern and northern China. There were differences between 2020/2021 and 2021/2022 nonepidemic seasons in northern (t=3.796, p<0.001) and southern China (t=3.902, p<0.001). There was no difference between 2020/2021 and 2021/2022 epidemic seasons in southern and northern China. During NPI implementation and COVID-19 vaccination stage, influenza infections were reduced by 68.97% in northern China compared to predicted value at the 6th week of 2022, and a 66.23% reduction was observed in southern China at the 4th week compared to the predicted value. 

## 4. Discussion

To our knowledge, this is the first study to explore the long-term relationship between Chinese prevention and control measures and influenza during the COVID-19 pandemic, especially for the impact under different prevention and control levels. Our result showed that China influenza activity was affected by the COVID-19 pandemic and different prevention and control measures during 2020-2022. The weekly ILI% observed value was generally lower than the predicted value in southern and northern China during 2020-2022. The median of impact index of weekly ILI% was 15.11% in north and 22.37% in south in 2020/2021 season and decreased significantly to 2.20% in north and 3.89% in south in 2021/2022 season.

Our results showed that influenza infection in southern and northern China had obvious seasonal characteristics from 2010 to 2019, while no obvious seasonal epidemic characteristics and no obvious peak were observed in 2020/2021 season after COVID-19 outbreak. The curves of weekly ILI% observed value during 2020-2022 were flat and lower than those during the same period in 2010-2019. COVID-19 outbreak changed the epidemic trend and characteristics of influenza in China. Our results were consistent with previous studies. Japan was one of the first countries to report a significant decrease in influenza activity in 2020 [19]. Sharp declines of influenza infections were observed in the United States and several countries in the Northern Hemisphere in early 2020 [11, 20]. Similar observations were reported in Australia, Chile, South Africa, and New Zealand during their influenza season in 2020 [9]. Because of the same host between influenza virus and SARS-CoV-2, the possible effect of “viral interference” on the prevalence and severity of the respective infections should be considered when influenza viruses and SARS-CoV-2 are cocirculated [5]. Competition between those two respiratory viruses that occurred in the human upper respiratory epithelium might lead to COVID-19 becoming the dominant virus, resulting in lower infection rates for influenza viruses [10].

China influenza activity was affected under different COVID-19 prevention and control levels during 2020-2022. Instead of assessing the specific effect of NPIs on influenza epidemics, we analyzed the epidemiology and seasonal patterns of influenza based on the timeline of COVID-19 NPIs implemented. At the beginning of 2020, influenza and COVID-19 outbreaks existed at the same time [11]. Then, there were low influenza epidemic levels and continued to decline in stage IV. In May 2020, the ongoing prevention and control stage started with an increasing influenza activity. As the prevention and control stage progressed, local policies varied depending on the severity of the COVID-19 outbreak, and it has been suggested that implementation of NPIs responding to community transmission of COVID-19 was associated with a reduction in seasonal influenza activity [21, 22]. Due to the same route and mode of transmission between influenza virus and SARS-CoV-2, the decrease in seasonal influenza activity could be attributed to extensive and rigorous implementation of NPIs [9].

The weekly ILI% observed value was generally lower than the predicted value in southern and northern China during 2020-2022, while a higher discrete degree was observed in 2021/2022 season. From August 2021, China’s epidemic prevention and control stage entered the “dynamic COVID-zero” phase of whole-chain precise prevention and control. The peak of winter and spring epidemics occurred in both southern and northern China. After COVID-19 prevention and control entered the dynamic COVID-zero stage, the local epidemic in China was sporadic, and social and economic life quickly returned to normal [15]. Meanwhile, resurgence of other respiratory viruses whose activity was suppressed under COVID-19 measures in 2020-2021 was observed around the world [23], with the possible recovery of influenza virus. With the increase in the low flu season number and the NPI implementation during 2020-2022, people are missing opportunities for enhanced immunity during a low influenza season [23]. Thus, populations with lower levels of immunity, such as the young children and the elderly individuals, are likely to experience more widespread illness and possibly a more severe burden when influenza viruses reemerge.

With the implementation of the COVID-19 NPIs, the median impact index of weekly ILI% was 22.37% in southern China and 15.11% in north in 2020/2021 season. The impact index also reached the peak in 2020/2021 surveillance year at the midseason of epidemic, which indicated that influenza infection decreased significantly in epidemic season and suggested a positive influence of COVID-19 prevention measures on influenza prevention in China. Studies suggested that without NPIs for COVID-19, influenza activity in China and the US was likely to remain high activity during the 2020 pandemic season [11]. Many studies have proven that NPI measures have a positive impact on prevention of influenza and other infectious diseases [24, 25], which is consistent with our results. Different impact indexes were also observed between southern and northern China. In addition to the different influenza epidemic characteristics, NPI implementation across the country faced the various conditions and specific challenges in different geographic regions during the pandemic. Clusters of outbreaks in the south and the north during the COVID-19 pandemic may affect the implementation of NPIs in different provinces, resulting in different prevention and control influences between southern and northern China.

However, the positive influence of NPIs on influenza prevention in the 2020/2021 season was observed to attenuate in the subsequent influenza seasons. The median impact index of weekly ILI% in 2021/2022 season was much lower than that in 2020/2021 season. Such change might be explained by resumed socioeconomic activities nationwide under COVID-19-related NPIs during the normal stage [26]. In addition, the compliance and effect of measures, such as wearing masks and maintaining social distancing, varied among individuals [27]. WHO introduced the term “pandemic fatigue” to indicate “a natural and expected reaction” of people during the COVD-19 pandemic [28], which might affect the implementation of NPIs. Meanwhile, influenza virus was sustained circulating in the population, which allowed for the resurgence of influenza epidemic [27]. A previous study showed higher ILI% and higher influenza positive rate in China during 2021/2022 season [29]. The same trend has been mentioned in other countries’ studies [30, 31].

There are several limitations in our study. First, in the time series model, only calendar years was controlled as the confounder. Whether circulating viruses matched the vaccine viruses and the influenza vaccination rate are two other confounders should be considered. Previous studies showed the influenza B/Victoria lineage virus was the predominant virus in China in the 2020/2021 and 2021/2022 seasons during the COVID-19 pandemic [29]. Recommended composition of influenza virus vaccines for use in the 2020-2022 northern hemisphere influenza season by WHO contained the same kind of virus (B/Washington/02/2019 (B/Victoria lineage)-like virus) [32, 33]. Therefore, the circulating influenza virus matched influenza vaccines between 2020 and 2022, which is not likely to affect our results. Influenza vaccination rate was not taken into account due to the fact that we cannot get further information, which might influence the results of this study. Second, our study used ILI% as an indicator for statistical analysis and description, which was based on the analysis of ILI monitoring data of symptoms. We were unable to obtain accurate data on the total visit number of outpatient or emergency department and specific data for each province to quantify the impact of these factors on our results. However, because of the complicated medical procedures during the pandemic, the number of visits in clinic and hospital might have been reduced [11], which may underestimate the influenza epidemic situation. Third, due to the sizeable fluctuation, our method may have limitations in describing the trend causing the large confidence intervals of EAPC in some weeks and might affect our prediction results to a certain extent. We did not make further correction in the calculation of EAPC, and we believe that the impact on the results was small.

In summary, there was a significant decrease in reported ILI in China during the 2020-2022 COVID-19 pandemic, particularly in winter and spring. Reduction of influenza virus infection might relate to everyday Chinese public health COVID-19 interventions. While increasing influenza vaccine coverage, influenza surveillance should continue to be strengthened, and a more comprehensive surveillance system for influenza virus and SARS-CoV-2 should be established. More studies should be performed to confirm our results, which can guide targeted and effective prevention strategies in the future.

## Data Availability

National Influenza Surveillance Network data are available from https://ivdc.chinacdc.cn/cnic/.

## Supplementary Materials

Supplementary Materials Table S1: weekly ILI% average value from 2010 to 2019, EAPC and DW statistic of sentinel hospitals in southern and northern China. Table S2: weekly ILI% predicted value, observed value, and impact index of sentinel hospitals in northern China from 2020 to 2022. Table S3: weekly ILI% predicted value, observed value and impact index of sentinel hospitals in southern China from 2020 to 2022.
