# Supplementary material for: Impact of COVID-19 Prevention and Control on the Influenza Epidemic in China: A Time Series Study
Source: Health Data Sci. 2022 Nov 1;2022:9830159. doi: 10.34133/2022/9830159 (PMC10880177; doi:10.34133/2022/9830159)
Supplement: Supplementary Materials — Table S1: weekly ILI% average value from 2010 to 2019, EAPC and DW statistic of sentinel hospitals in southern and northern China. Table S2: weekly ILI% predicted value, observed value, and impact index of sentinel hospitals in northern China from 2020 to 2022. Table S3: weekly ILI% predicted value, observed value and impact index of sentinel hospitals in southern China from 2020 to 2022. [file 9830159.f1.docx]

Table S1 Weekly ILI% average value from 2010 to 2019, EAPC and DW-statistic of sentinel hospitals in southern and northern China.

|  | South | | |  | North | | |
| --- | --- | --- | --- | --- | --- | --- | --- |
| Week | 2010-2019 average value（%） | EAPC（CIs） | DW-statistic |  | 2010-2019 average value（%） | EAPC（CIs） | DW-statistic |
| 14 | 3.4 | 2.21(-0.48 - 4.97) | 2.21 |  | 3.2 | -1.2(-5.29 - 3.07) | 2.33 |
| 15 | 3.3 | 1.62(-0.85 - 4.15) | 1.86 |  | 3.0 | -1.45(-5.94 - 3.26) | 2.39 |
| 16 | 3.4 | 1.74(-0.73 - 4.28) | 1.53 |  | 3.0 | -1.87(-6.9 - 3.43) | 2.27 |
| 17 | 3.3 | 1.61(-1.15 - 4.45) | 1.52 |  | 3.0 | -2.05(-6.68 - 2.81) | 2.55 |
| 18 | 3.5 | 1.01(-1.61 - 3.7) | 1.58 |  | 3.3 | -3.37(-9.69 - 3.39) | 2.52 |
| 19 | 3.4 | 1.46(-1.16 - 4.16) | 1.00 |  | 2.9 | -3.79(-7.79 - 0.38) | 2.47 |
| 20 | 3.5 | 1.34(-0.97 - 3.7) | 0.94 |  | 3.0 | -4.05(-8.52 - 0.65) | 2.61 |
| 21 | 3.5 | 1.73(-0.52 - 4.03) | 0.73 |  | 2.8 | -3.92(-7.83 - 0.15) | 2.41 |
| 22 | 3.6 | 1.99(0.08 - 3.93) | 1.39 |  | 2.9 | -4.68(-7.93 - -1.3) | 2.09 |
| 23 | 3.6 | 3.76(1.56 - 6.02) | 1.61 |  | 2.9 | -4.72(-7.65 - -1.71) | 1.83 |
| 24 | 3.7 | 1.01(-1.32 - 3.39) | 1.91 |  | 2.9 | -6.36(-9.61 - -2.99) | 2.55 |
| 25 | 3.7 | 1.18(-0.68 - 3.06) | 2.27 |  | 2.9 | -5.23(-7.49 - -2.91) | 2.22 |
| 26 | 3.6 | 1.05(-0.94 - 3.08) | 2.48 |  | 2.9 | -5.91(-8.62 - -3.12) | 2.28 |
| 27 | 3.6 | 0.43(-1.99 - 2.9) | 2.92 |  | 2.9 | -5.51(-7.89 - -3.07) | 2.60 |
| 28 | 3.5 | -0.02(-2.49 - 2.52) | 3.18 |  | 2.8 | -5.21(-7.18 - -3.19) | 3.10 |
| 29 | 3.5 | -0.38(-2.95 - 2.27) | 3.44 |  | 2.8 | -5.67(-7.72 - -3.57) | 2.65 |
| 30 | 3.5 | -1.29(-4.13 - 1.64) | 3.03 |  | 2.8 | -5.29(-7.16 - -3.38) | 2.28 |
| 31 | 3.4 | -1.43(-3.94 - 1.15) | 2.97 |  | 2.7 | -5.6(-7.91 - -3.22) | 2.40 |
| 32 | 3.3 | -1.57(-4.34 - 1.27) | 2.72 |  | 2.7 | -5.54(-7.76 - -3.27) | 2.74 |
| 33 | 3.2 | -2.06(-4.95 - 0.92) | 2.07 |  | 2.7 | -5.51(-8.18 - -2.77) | 2.74 |
| 34 | 3.0 | -1.83(-5.01 - 1.46) | 2.09 |  | 2.6 | -5.48(-8.08 - -2.81) | 2.77 |
| 35 | 3.0 | -2.32(-5.5 - 0.97) | 2.00 |  | 2.6 | -5.69(-7.81 - -3.53) | 2.48 |
| 36 | 3.0 | -2.31(-5.28 - 0.75) | 1.52 |  | 2.7 | -6.1(-8.87 - -3.25) | 2.07 |
| 37 | 3.1 | -1.13(-4.51 - 2.38) | 1.62 |  | 2.8 | -6.36(-9.67 - -2.93) | 1.92 |
| 38 | 3.0 | -1.31(-4.85 - 2.37) | 1.45 |  | 2.7 | -5.66(-8.23 - -3.01) | 2.37 |
| 39 | 2.8 | 1.05(-1.66 - 3.83) | 1.15 |  | 2.6 | -4.67(-6.97 - -2.31) | 2.43 |
| 40 | 3.2 | 0.53(-2.36 - 3.49) | 1.17 |  | 3.2 | -2.95(-6.29 - 0.52) | 1.83 |
| 41 | 2.8 | 0.42(-3.02 - 3.98) | 1.64 |  | 2.6 | -3.87(-5.69 - -2.02) | 2.57 |
| 42 | 2.7 | -0.66(-3.51 - 2.27) | 1.03 |  | 2.7 | -2.81(-4.92 - -0.65) | 2.47 |
| 43 | 2.7 | 0.12(-2.74 - 3.06) | 1.16 |  | 2.8 | -3.37(-5.64 - -1.05) | 2.55 |
| 44 | 2.8 | 0.57(-2.28 - 3.51) | 1.42 |  | 2.9 | -3.61(-5.58 - -1.6) | 2.69 |
| 45 | 2.7 | 0.18(-3.22 - 3.7) | 1.33 |  | 2.8 | -2.59(-4.92 - -0.22) | 1.41 |
| 46 | 2.7 | 1.16(-1.91 - 4.33) | 1.17 |  | 3.0 | -2.75(-5.6 - 0.18) | 2.48 |
| 47 | 2.8 | 2.02(-0.92 - 5.04) | 1.49 |  | 3.2 | -2.02(-5.02 - 1.07) | 2.23 |
| 48 | 2.9 | 3.39(0.11 - 6.78) | 1.27 |  | 3.3 | -0.93(-3.9 - 2.14) | 2.31 |
| 49 | 3.0 | 3.67(-0.23 - 7.72) | 1.68 |  | 3.5 | 0.03(-3.45 - 3.63) | 2.45 |
| 50 | 3.1 | 5.71(0.38 - 11.33) | 1.43 |  | 3.8 | 2.05(-2.41 - 6.71) | 2.49 |
| 51 | 3.4 | 6.08(1.18 - 11.21) | 1.57 |  | 3.9 | 3.04(-2.13 - 8.49) | 2.53 |
| 52 | 3.6 | 7.72(2.54 - 13.16) | 1.84 |  | 4.1 | 3.05(-1.71 - 8.03) | 2.31 |
| 1 | 4.0 | 8.28(2.16 - 14.77) | 2.34 |  | 4.6 | 3.29(-1.53 - 8.34) | 1.49 |
| 2 | 4.0 | 7.94(1.91 - 14.34) | 1.77 |  | 4.4 | 2.64(-1.95 - 7.44) | 1.33 |
| 3 | 4.0 | 7.51(1.08 - 14.35) | 1.94 |  | 4.4 | 1.63(-2.2 - 5.61) | 1.39 |
| 4 | 4.2 | 7.72(0.68 - 15.26) | 1.35 |  | 4.6 | 3.48(-1.78 - 9.03) | 1.21 |
| 5 | 4.2 | 6.89(-0.33 - 14.63) | 1.29 |  | 4.7 | 3.36(-3.08 - 10.23) | 1.43 |
| 6 | 4.0 | 7.25(0.15 - 14.86) | 2.20 |  | 4.4 | 5.26(-1.09 - 12.01) | 2.39 |
| 7 | 3.5 | 4.56(-1.2 - 10.65) | 1.84 |  | 3.8 | 2.66(-3.35 - 9.03) | 2.21 |
| 8 | 3.4 | 2.27(-2.82 - 7.63) | 2.08 |  | 3.5 | 0.63(-3.61 - 5.07) | 2.56 |
| 9 | 3.2 | 3.08(-0.61 - 6.9) | 1.71 |  | 3.1 | 0.06(-2.72 - 2.91) | 2.17 |
| 10 | 3.1 | 2.22(-1.75 - 6.35) | 2.24 |  | 3.0 | -1.94(-5.1 - 1.32) | 2.10 |
| 11 | 3.2 | 2.04(-2.3 - 6.57) | 2.57 |  | 3.1 | -1.43(-5.34 - 2.63) | 2.49 |
| 12 | 3.2 | 1.44(-2.71 - 5.77) | 2.55 |  | 3.0 | 0.07(-4.22 - 4.55) | 2.43 |
| 13 | 3.2 | 1.45(-3.02 - 6.13) | 2.28 |  | 2.9 | 0.25(-3.82 - 4.49) | 2.50 |

Table S2 Weekly ILI% predicted value, observed value and impact index of sentinel hospitals in northern China from 2020 to 2022.

| Week | 2020/2021 season | | |  | 2021/2022 season | | |
| --- | --- | --- | --- | --- | --- | --- | --- |
|  | Predicted value | Observed value | Impact index |  | Predicted value | Observed value | Impact index |
| 14 | 3.85(3.69 - 4.02) | 2.1 | 45.50 |  | 3.85(3.69 - 4.02) | 2.1 | 44.84 |
| 15 | 3.65(3.48 - 3.82) | 1.9 | 47.90 |  | 3.65(3.48 - 3.82) | 2.2 | 38.78 |
| 16 | 3.53(3.35 - 3.72) | 1.9 | 46.22 |  | 3.53(3.35 - 3.72) | 2.3 | 33.66 |
| 17 | 3.23(3.08 - 3.39) | 1.8 | 44.31 |  | 3.23(3.08 - 3.39) | 2.4 | 24.19 |
| 18 | 3.09(2.89 - 3.31) | 1.9 | 38.55 |  | 3.09(2.89 - 3.31) | 2.5 | 16.33 |
| 19 | 2.5(2.4 - 2.61) | 1.9 | 24.05 |  | 2.5(2.4 - 2.61) | 2.4 | 0.28 |
| 20 | 2.4(2.29 - 2.52) | 1.9 | 20.80 |  | 2.4(2.29 - 2.52) | 2.7 | -17.30 |
| 21 | 2.4(2.3 - 2.5) | 1.9 | 20.90 |  | 2.4(2.3 - 2.5) | 2.8 | -21.33 |
| 22 | 2.29(2.21 - 2.37) | 1.9 | 16.95 |  | 2.29(2.21 - 2.37) | 3.0 | -37.56 |
| 23 | 2.48(2.4 - 2.56) | 2.0 | 19.26 |  | 2.48(2.4 - 2.56) | 3.1 | -31.35 |
| 24 | 2.15(2.08 - 2.23) | 2.2 | -2.14 |  | 2.15(2.08 - 2.23) | 2.9 | -43.78 |
| 25 | 2.18(2.13 - 2.23) | 2.4 | -10.10 |  | 2.18(2.13 - 2.23) | 2.7 | -30.70 |
| 26 | 2.26(2.19 - 2.33) | 2.2 | 2.57 |  | 2.26(2.19 - 2.33) | 2.6 | -22.37 |
| 27 | 2.27(2.21 - 2.33) | 2.0 | 11.81 |  | 2.27(2.21 - 2.33) | 2.4 | -12.01 |
| 28 | 2.18(2.13 - 2.23) | 1.9 | 12.85 |  | 2.18(2.13 - 2.23) | 2.4 | -16.12 |
| 29 | 2.08(2.03 - 2.12) | 1.8 | 13.27 |  | 2.08(2.03 - 2.12) | 2.4 | -22.60 |
| 30 | 2.18(2.14 - 2.22) | 2.0 | 8.18 |  | 2.18(2.14 - 2.22) | 2.4 | -16.34 |
| 31 | 2.08(2.03 - 2.13) | 2.0 | 3.70 |  | 2.08(2.03 - 2.13) | 2.3 | -17.31 |
| 32 | 1.98(1.94 - 2.03) | 1.9 | 4.21 |  | 1.98(1.94 - 2.03) | 2.2 | -17.42 |
| 33 | 1.98(1.93 - 2.04) | 1.9 | 4.24 |  | 1.98(1.93 - 2.04) | 2.0 | -6.68 |
| 34 | 1.89(1.84 - 1.94) | 1.7 | 10.07 |  | 1.89(1.84 - 1.94) | 1.7 | 4.85 |
| 35 | 1.89(1.84 - 1.93) | 1.8 | 4.57 |  | 1.89(1.84 - 1.93) | 1.6 | 10.05 |
| 36 | 1.97(1.91 - 2.03) | 2.0 | -1.43 |  | 1.97(1.91 - 2.03) | 1.8 | 2.79 |
| 37 | 2.15(2.08 - 2.23) | 2.0 | 7.14 |  | 2.15(2.08 - 2.23) | 2.1 | -4.13 |
| 38 | 2.08(2.02 - 2.13) | 2.1 | -1.18 |  | 2.08(2.02 - 2.13) | 2.2 | -12.35 |
| 39 | 2.19(2.14 - 2.25) | 2.2 | -0.34 |  | 2.19(2.14 - 2.25) | 2.2 | -5.25 |
| 40 | 2.81(2.72 - 2.92) | 2.5 | 11.18 |  | 2.73(2.55 - 2.93) | 2.2 | 19.46 |
| 41 | 2.11(2.07 - 2.16) | 2.4 | -13.48 |  | 2.03(1.96 - 2.11) | 2.0 | 1.62 |
| 42 | 2.33(2.28 - 2.38) | 2.2 | 5.69 |  | 2.27(2.17 - 2.37) | 2.3 | -1.45 |
| 43 | 2.32(2.26 - 2.37) | 2.3 | 0.82 |  | 2.24(2.14 - 2.35) | 2.6 | -16.03 |
| 44 | 2.41(2.36 - 2.46) | 2.4 | 0.41 |  | 2.32(2.23 - 2.42) | 2.6 | -11.93 |
| 45 | 2.44(2.38 - 2.49) | 2.5 | -2.66 |  | 2.37(2.26 - 2.49) | 2.7 | -13.83 |
| 46 | 2.53(2.45 - 2.6) | 2.5 | 1.12 |  | 2.46(2.32 - 2.61) | 2.8 | -13.88 |
| 47 | 2.84(2.75 - 2.93) | 2.5 | 12.01 |  | 2.78(2.62 - 2.96) | 2.8 | -0.58 |
| 48 | 3.27(3.17 - 3.37) | 2.4 | 26.59 |  | 3.24(3.05 - 3.44) | 3.0 | 7.38 |
| 49 | 3.7(3.57 - 3.83) | 2.6 | 29.75 |  | 3.7(3.45 - 3.97) | 3.3 | 10.86 |
| 50 | 4.8(4.59 - 5.02) | 2.7 | 43.71 |  | 4.89(4.48 - 5.35) | 3.5 | 28.50 |
| 51 | 5.46(5.19 - 5.75) | 2.8 | 48.73 |  | 5.63(5.08 - 6.24) | 3.9 | 30.69 |
| 52 | 5.67(5.41 - 5.94) | 2.9 | 48.83 |  | 5.84(5.31 - 6.42) | 4.1 | 29.80 |
| 1 | 6.09(5.81 - 6.39) | 2.5 | 58.98 |  | 6.29(5.72 - 6.93) | 4.0 | 36.45 |
| 2 | 5.44(5.2 - 5.69) | 2.7 | 50.36 |  | 5.58(5.1 - 6.12) | 3.7 | 33.73 |
| 3 | 4.57(4.4 - 4.75) | 2.6 | 43.15 |  | 4.65(4.3 - 5.02) | 3.1 | 33.30 |
| 4 | 7.04(6.68 - 7.41) | 2.4 | 65.89 |  | 7.28(6.56 - 8.08) | 2.6 | 64.29 |
| 5 | 8.79(8.24 - 9.37) | 2.0 | 77.24 |  | 9.08(7.99 - 10.33) | 3.5 | 61.46 |
| 6 | 6.74(6.33 - 7.17) | 2.6 | 61.40 |  | 7.09(6.26 - 8.03) | 2.2 | 68.97 |
| 7 | 5.54(5.22 - 5.89) | 2.0 | 63.92 |  | 5.69(5.04 - 6.42) | 1.9 | 66.61 |
| 8 | 4.43(4.24 - 4.62) | 1.7 | 61.61 |  | 4.46(4.09 - 4.86) | 1.9 | 57.36 |
| 9 | 3.5(3.4 - 3.6) | 1.6 | 54.31 |  | 3.5(3.31 - 3.71) | 2.2 | 37.22 |
| 10 | 2.75(2.66 - 2.84) | 1.8 | 34.44 |  | 2.69(2.52 - 2.87) | 2.5 | 7.15 |
| 11 | 2.46(2.37 - 2.57) | 1.9 | 22.89 |  | 2.43(2.24 - 2.63) | 2.4 | 1.19 |
| 12 | 2.4(2.3 - 2.51) | 2.1 | 12.56 |  | 2.4(2.2 - 2.62) | 2.2 | 8.46 |
| 13 | 2.31(2.21 - 2.4) | 2.2 | 4.59 |  | 2.31(2.13 - 2.51) | 2.0 | 13.48 |

Table S3 Weekly ILI% predicted value, observed value and impact index of sentinel hospitals in southern China from 2020 to 2022.

|  | 2020/2021 season | | |  | 2021/2022 season | | |
| --- | --- | --- | --- | --- | --- | --- | --- |
| Week | Predicted value | Observed value | Impact index |  | Predicted value | Observed value | Impact index |
| 14 | 4.29(4.18 - 4.41) | 2.2 | 48.75 |  | 4.39(4.16 - 4.63) | 3.0 | 31.62 |
| 15 | 4.06(3.97 - 4.17) | 2.3 | 43.42 |  | 4.13(3.93 - 4.34) | 3.1 | 24.95 |
| 16 | 4.27(4.17 - 4.38) | 2.3 | 46.18 |  | 4.35(4.14 - 4.57) | 3.4 | 21.80 |
| 17 | 4.27(4.15 - 4.39) | 2.4 | 43.76 |  | 4.34(4.1 - 4.58) | 3.7 | 14.67 |
| 18 | 4.34(4.23 - 4.46) | 2.6 | 40.14 |  | 4.39(4.16 - 4.62) | 3.9 | 11.11 |
| 19 | 4.06(3.95 - 4.17) | 2.7 | 33.47 |  | 4.12(3.91 - 4.34) | 3.7 | 10.15 |
| 20 | 4.15(4.06 - 4.25) | 2.7 | 35.01 |  | 4.21(4.02 - 4.41) | 4.0 | 4.99 |
| 21 | 4.27(4.18 - 4.37) | 2.9 | 32.13 |  | 4.35(4.16 - 4.55) | 4.2 | 3.37 |
| 22 | 4.18(4.1 - 4.26) | 2.9 | 30.65 |  | 4.26(4.11 - 4.43) | 4.3 | -0.83 |
| 23 | 4.77(4.67 - 4.88) | 3.0 | 37.15 |  | 4.95(4.74 - 5.17) | 4.4 | 11.16 |
| 24 | 4.24(4.14 - 4.34) | 3.3 | 22.21 |  | 4.29(4.09 - 4.49) | 4.2 | 1.99 |
| 25 | 4.05(3.97 - 4.12) | 3.3 | 18.46 |  | 4.09(3.95 - 4.25) | 4.0 | 2.31 |
| 26 | 4.04(3.96 - 4.12) | 3.2 | 20.83 |  | 4.08(3.93 - 4.25) | 3.8 | 6.96 |
| 27 | 3.92(3.82 - 4.01) | 2.8 | 28.51 |  | 3.93(3.75 - 4.13) | 3.7 | 5.93 |
| 28 | 3.5(3.41 - 3.59) | 2.7 | 22.84 |  | 3.5(3.33 - 3.68) | 3.6 | -2.89 |
| 29 | 3.59(3.49 - 3.68) | 2.7 | 24.72 |  | 3.57(3.39 - 3.77) | 3.5 | 2.04 |
| 30 | 3.36(3.26 - 3.46) | 2.6 | 22.53 |  | 3.31(3.12 - 3.51) | 3.3 | 0.39 |
| 31 | 3.25(3.17 - 3.34) | 2.7 | 17.00 |  | 3.21(3.05 - 3.38) | 3.2 | 0.20 |
| 32 | 3.25(3.16 - 3.34) | 2.7 | 16.87 |  | 3.2(3.02 - 3.38) | 3.0 | 6.16 |
| 33 | 2.94(2.85 - 3.03) | 2.7 | 8.11 |  | 2.88(2.71 - 3.06) | 2.8 | 2.70 |
| 34 | 2.95(2.85 - 3.04) | 2.6 | 11.72 |  | 2.89(2.71 - 3.09) | 2.6 | 10.08 |
| 35 | 2.74(2.65 - 2.83) | 2.7 | 1.28 |  | 2.67(2.5 - 2.85) | 2.7 | -1.06 |
| 36 | 2.83(2.75 - 2.92) | 3.1 | -9.42 |  | 2.77(2.6 - 2.94) | 3.1 | -12.01 |
| 37 | 3.36(3.25 - 3.48) | 3.7 | -10.06 |  | 3.32(3.1 - 3.56) | 3.5 | -5.30 |
| 38 | 3.16(3.04 - 3.28) | 3.9 | -23.49 |  | 3.12(2.9 - 3.35) | 3.8 | -21.91 |
| 39 | 3.23(3.15 - 3.32) | 3.4 | -5.15 |  | 3.27(3.09 - 3.45) | 3.5 | -7.12 |
| 40 | 3.52(3.42 - 3.62) | 3.6 | -2.32 |  | 3.54(3.34 - 3.75) | 3.4 | 3.87 |
| 41 | 2.81(2.72 - 2.91) | 3.3 | -17.36 |  | 2.82(2.63 - 3.03) | 2.7 | 4.38 |
| 42 | 2.88(2.8 - 2.97) | 3.1 | -7.61 |  | 2.86(2.7 - 3.03) | 3.0 | -4.83 |
| 43 | 3(2.92 - 3.09) | 3.3 | -9.87 |  | 3.01(2.84 - 3.19) | 3.5 | -16.39 |
| 44 | 3.12(3.03 - 3.21) | 3.6 | -15.47 |  | 3.14(2.96 - 3.32) | 3.5 | -11.63 |
| 45 | 3.11(3 - 3.21) | 3.6 | -15.92 |  | 3.11(2.9 - 3.33) | 3.4 | -9.27 |
| 46 | 3.24(3.14 - 3.34) | 3.8 | -17.39 |  | 3.27(3.08 - 3.48) | 3.4 | -3.82 |
| 47 | 3.47(3.37 - 3.57) | 3.7 | -6.67 |  | 3.54(3.34 - 3.75) | 3.4 | 3.91 |
| 48 | 3.62(3.5 - 3.74) | 3.5 | 3.28 |  | 3.74(3.51 - 3.99) | 3.8 | -1.56 |
| 49 | 4.15(3.99 - 4.31) | 3.4 | 18.01 |  | 4.3(3.98 - 4.64) | 3.9 | 9.27 |
| 50 | 5.5(5.22 - 5.79) | 3.5 | 36.33 |  | 5.81(5.24 - 6.45) | 3.9 | 32.89 |
| 51 | 5.94(5.67 - 6.23) | 3.4 | 42.76 |  | 6.3(5.73 - 6.93) | 4.0 | 36.52 |
| 52 | 6.68(6.36 - 7.02) | 3.5 | 47.59 |  | 7.19(6.52 - 7.94) | 4.0 | 44.40 |
| 1 | 7.15(6.74 - 7.57) | 3.4 | 52.42 |  | 7.74(6.89 - 8.69) | 4.3 | 44.43 |
| 2 | 6.58(6.22 - 6.97) | 3.5 | 46.84 |  | 7.11(6.33 - 7.97) | 3.9 | 45.13 |
| 3 | 5.48(5.16 - 5.83) | 3.4 | 37.99 |  | 5.9(5.21 - 6.67) | 3.5 | 40.63 |
| 4 | 7.97(7.45 - 8.53) | 3.0 | 62.37 |  | 8.59(7.5 - 9.83) | 2.9 | 66.23 |
| 5 | 8.55(7.97 - 9.17) | 2.6 | 69.59 |  | 9.14(7.95 - 10.51) | 3.9 | 57.33 |
| 6 | 6.11(5.71 - 6.55) | 3.2 | 47.66 |  | 6.56(5.72 - 7.52) | 2.7 | 58.82 |
| 7 | 4.81(4.54 - 5.09) | 2.7 | 43.86 |  | 5.03(4.49 - 5.63) | 2.6 | 48.30 |
| 8 | 3.68(3.5 - 3.87) | 2.2 | 40.25 |  | 3.77(3.4 - 4.17) | 3.1 | 17.68 |
| 9 | 3.4(3.28 - 3.53) | 2.4 | 29.45 |  | 3.51(3.26 - 3.77) | 4.0 | -14.08 |
| 10 | 2.96(2.85 - 3.08) | 2.4 | 19.04 |  | 3.03(2.8 - 3.28) | 4.0 | -32.01 |
| 11 | 2.86(2.74 - 2.98) | 2.8 | 2.00 |  | 2.92(2.67 - 3.18) | 4.3 | -47.50 |
| 12 | 2.74(2.63 - 2.86) | 2.9 | -5.88 |  | 2.78(2.56 - 3.02) | 3.7 | -33.17 |
| 13 | 2.43(2.33 - 2.55) | 3.0 | -23.21 |  | 2.47(2.26 - 2.7) | 3.6 | -45.73 |
